# Supplementary material for: Adverse event profile differences among long-acting gonadotropin-releasing hormone analogs: A real-world, pharmacovigilance study
Source: PLoS One. 2025 Jul 11;20(7):e0327842. doi: 10.1371/journal.pone.0327842 (PMC12250665; doi:10.1371/journal.pone.0327842)
Supplement: S1 Table — (DOCX) [file pone.0327842.s001.docx]

**S1 Table The corresponding algorithms, equations and criteria**

| **Algorithms** | **Equation** | **Criteria** |
| --- | --- | --- |
| ROR | ROR = $\frac{\frac{\mathrm{DE}}{\mathrm{De}}}{\frac{\mathrm{dE}}{\mathrm{de}}}$  95%CI =$e^{\ln\left（ \mathrm{ROR} \right） \pm1.96\sqrt{\frac{1}{\mathrm{DE}}+\frac{1}{\mathrm{De}}+\frac{1}{\mathrm{dE}}+\frac{1}{\mathrm{de}}}}$  $\chi_{Yates}^{2}$= N * $\frac{\left( \left\vert DE*de- dE*De \right\vert- \frac{N}{2} \right)^{2}}{D*d*E*e}$ | 1. DE≥3; 2. ROR≥2; 3. χ^2^_Yates_ ≥ 3.841; 4. 95% CI lower-bound>1. |
| BCPNN | IC=$\log_{2} \frac{DE+0.5}{\mathrm{DE}_{\exp}+0.5}$  $\mathrm{DE}_{\exp}$=$\frac{\left( DE+dE \right)*\left( DE+De \right)}{\left( DE+dE+De+de \right)}$  IC_025_=IC-3.3*$\left( DE+0.5 \right)^{-\frac{1}{2}}$- 2*$\left( DE+0.5 \right)^{-\frac{3}{2}}$ | 1. DE≥3; 2. Weak signal: 0<IC_025_≤1.5; 3. Medium signal: 1.5<IC_025_≤3; 4. Strong signal: IC_025_>3. |

ROR, Reporting Odds Ratio; BCPNN, Bayesian Confidence Propagation Neural Network; IC, Information Component; IC025, the lower limit of Information Component.

Noren, G. N., Bate, A., Orre, R., & Edwards, I. R. (2006). Extending the methods used to screen the WHO drug safety database towards analysis of complex associations and improved accuracy for rare events. Stat Med, 25(21), 3740-3757. Retrieved from https://www.ncbi.nlm.nih.gov/pubmed/16381072. doi:10.1002/sim.2473
