# Supplementary material for: Adverse event profile differences among long-acting gonadotropin-releasing hormone analogs: A real-world, pharmacovigilance study
Source: PLoS One. 2025 Jul 11;20(7):e0327842. doi: 10.1371/journal.pone.0327842 (PMC12250665; doi:10.1371/journal.pone.0327842)
Supplement: Attachment — Submitted filename: Response to Reviewers.pdf [file pone.0327842.s007.pdf]

Dear editor and reviewers,

First of all, we sincerely appreciate your review of our manuscript. Your valuable feedback holds significant importance in guiding our research work. We have thoroughly considered the review comments and made corresponding revisions to the manuscript according to these suggestions. Next, we respond to the comments made by the reviewers one by one.

Reviewer #1: Chen et al entitle "Adverse Event Profile Differences among Long-acting Gonadotropin-releasing hormone analogs: A Real-world, Pharmacovigilance Study" clearly reported that long-acting GnRHa could be adverse drug for prostate cancer, breast cancer, endometriosis, uterine leiomyomas, and central precocious puberty.

Why are you using the FAERS database to study this drug, it should be that someone else has done this with this similar database similar methodology, so you can write cite in the INTRODUCTION section about some specific other similar studies, such as recommending a few (It is equivalent to saying that someone else has done this type of research using the FAERS database, and you can use this database to do research related to long-acting GnRHa as well):【1】Wang Y, Zhao B, Yang H, Wan Z. A real-world pharmacovigilance study of FDA adverse event reporting system events for sildenafil. Andrology. 2024 May;12(4):785-792. doi: 10.1111/andr.13533. Epub 2023 Sep 19. PMID: 37724699.【2】Zhao B, Fu Y, Cui S, Chen X, Liu S, Luo L. A real-world disproportionality analysis of Everolimus: data mining of the public version of FDA adverse event reporting system. Front Pharmacol. 2024 Mar 12;15:1333662. doi: 10.3389/fphar.2024.1333662. PMID: 38533254; PMCID: PMC10964017.【3】Yang H, Wan Z, Chen M, Zhang X, Cui W, Zhao B. A real-world data analysis of topotecan in the FDA Adverse Event Reporting System (FAERS) database. Expert Opin Drug Metab Toxicol. 2023 Apr;19(4):217-223. doi: 10.1080/17425255.2023.2219390. Epub 2023 May 30. PMID: 37243615.【4】Zhao B, Zhang X, Chen M, Wang Y. A real-world data analysis of acetylsalicylic acid in FDA Adverse Event Reporting System (FAERS) database. Expert Opin Drug Metab Toxicol. 2023 Jan-Jun;19(6):381-387. doi: 10.1080/17425255.2023.2235267. Epub 2023 Jul 12. PMID: 37421631.【5】Li, Jie, Zhao, Bin, Zhu, YongQing, Wu, Jibiao, Vitreoretinal Traction Syndrome, Nitrituria and Human Epidermal Growth Factor Receptor Negative Might Occur in the Aromatase-Inhibitor Anastrozole Treatment, International Journal of Clinical Practice, 2024, 5132916, 9 pages, 2024. https://doi.org/10.1155/2024/5132916【6】Zhong, C., Zheng, Q., Zhao, B., & Ren, T. (2024). A real-world pharmacovigilance study using disproportionality analysis of United States Food and Drug Administration Adverse Event Reporting System events for vinca alkaloids: comparing vinorelbine and Vincristine. Expert Opinion on Drug Safety, 23(11), 1427–1437. https://doi.org/10.1080/14740338.2024.2410436

Response: We have used these studies in the"Introduction"as examples of research methods provided in our research and have cited relevant references . Please refer to Page 4 in the Revised Manuscript with Track Changes for details.

Besides,

I have general suggestion is:

1) Can Author put a bar chart for the percentage of long-acting GnRHa uses in five country and hospitalization, death, life threating events, and disability.

Response: Figure 1 have shown it in pie chart form. Thank you for your insightful advice.

2) I also encourage to author please put the bar chart for table where author indicated that prostate

cancer, breast cancer, endometriosis, uterine leiomyomas, and central precocious puberty related adverse event for use of long-acting GnRHa.

Response: Figure 1 have shown it in pie chart form. Thank you for your insightful advice.

3) I also encourage the author please use 1 graphical representation of long-acting GnRHa and their adverse event (long-acting GnRHa affecting which organ in human body)

Response: Thank you for your suggestion.We have made the corresponding diagram and attached it at the end of this part of the "Introduction". Please refer to Page 4 in the Revised Manuscript with Track Changes for details.

Reviewer #2: The aim of the study was to investigate the adverse event (AE) profile differences of long-acting GnRHa reported by the US Food and Drug Administration Adverse Event Reporting System (FAERS).

Specific comments

Abstract

"Reproductive system and breast disorders possessed the greatest proportion in AEs of the five long-acting GnRHa." – review word choice "possessed"

Response: Thank you for your thoughtful comment regarding the diction in this sentence. We agree that "possessed" may convey unintended connotations of ownership in this scientific context. Following your suggestion, we have revised the sentence to: "Reproductive system and breast disorders accounted for the greatest proportion of adverse events among the five long-acting GnRHa formulations analyzed"(marked blue on page 2 in Revised Manuscript with Track Changes).

"Buserelin and histrelin demonstrated a higher percentage of AEs in the gastrointestinal and psychiatric disorders separately, which also exhibited a higher risk of important medical events in logistic regression analysis and the risk was lower in patients under 18 years old than those over 18 ." – Vary sentence structure and revise for clarity and accuracy.

Response: We sincerely appreciate the reviewer's valuable comments regarding the clarity and structure of this sentence. We have carefully revised the text to improve its readability and accuracy. The original sentence has been restructured to better convey the distinct adverse effect profiles of the two medications, clarify the statistical findings. The revised sentences are marked blue in the " Results "of the "Abstract" on page 2 in our Revised Manuscript with Track Changes.

Introduction

Paragraph 1

"exacerbates, a range of hormone-dependent diseases" - vague, identify the diseases, as the risk is not equal/comparable

"LHRH"- provide a detailed description of the abbreviation at first use

Response: We have attached an explanation of this English abbreviation in "Introduction" in our Revised Manuscript with Track Changes (blue font on page 3). Thank you for your careful check.

"puberty, endometriosis, adenomyosis etc.," – complete the list appropriately, not academic convention

Response: In order to meet the academic convention, we have removed "etc.".

“also for the prevention of chemotherapy-induced premature ovarian failure in cancer women[2-5].” – revise expression “cancer woman”

Response: Thank you for your valuable comment. We agree that the expression ‘cancer woman’ is not precise. We have revised it to ‘gynecological cancer’ to better reflect the intended meaning and improve clarity. In the Revised Manuscript with Track Changes, this modified phrase has been highlighted in blue on page 3.

Paragraph 2: Identify adverse reactions with appropriate type of GnRHa. Are these findings relevant to particular use/dosage of GnRHa administered?

Response: As we mentioned in limitations: The spontaneous reports within the FAERS database may suffer from quantitative biases and incomplete documentation. A large part of the data on the route and dosage of the five drugs is missing or unknown. Therefore, we believe that these data cannot support whether the route of administration or dose is associated with the reported AE. However, our future plan is to investigate this issue through meta-analysis or clinical data. Thank you for pointing out the direction for our future research. We have also added future research plans to conclusions. Please refer to Page 18 in the Revised Manuscript with Track Changes for details (marked with blue font).

“CPP” - provide a detailed description of the abbreviation at first use

Response: We are sorry for this mistake. We have abbreviated where CPP first appears and identified it in blue font (in “Background” of “Abstract”).

“hypertension (HTN)[10]” - is this an accepted abbreviation?

Response: HTN is the accepted abbreviation for hypertension. The abbreviation is correct. However, in order to make the sentence more clear, we kept "hypertension" and do not use its abbreviation.

“had been covered during the use of GnRHa” - not clear what this claim means, revise language

Response: We have carefully revised the text to improve its readability and accuracy. Please refer to Page 3 in the Revised Manuscript with Track Changes for details (marked with blue font).

“As one of the effective drugs for treating prostate cancer, the adverse reactions reported including life-threatening anaphylaxis[11], follicular mucinosis and mycosis-fungoides-like drug” - Not clear which drug/s the authors are implying here, provide details.

Response: The drug here also refers to long-acting GnRHa to provide a general overview of the common adverse reactions reported in various indications. It's that our description wasn't clear enough. To this end, we have revised the description of the previous part of this sentence, which is marked in blue font on page 3 in Revised Manuscript with Track Changes.

“…follicular mucinosis and mycosis-fungoides-like drug eruption[12], cardiovascular (CV)[13].”- revise for grammar and clarity, what aspect of cardiovascular does the authors imply here?

Response: It meaned that long-acting GnRHa was more likely to cause cardiovascular toxicity in the treatment of prostate cancer. Because of our handwriting error, we omitted the word toxic. We

have made the correction (blue font on page 3 in the Revised Manuscript with Track Changes). Thank you for your careful reminder.

Paragraph 3

“Therefore, we utilize FAERS to evaluate the safety characteristics of long-acting GnRHa and provide a practical and secure reference for their clinical applications.”- review for grammar, utilised/utilized (past tense)

Response: The tense of “utilize” has been changed to the past tense (marked with blue font on page 4 in the Revised Manuscript with Track Changes).

Materials and Methods

Data sources

“and therapeutic biologic products” – review grammar, “biological”

Response: In the Revised Manuscript with Track Changes, "biologic" has been modified to "biological" (marked with blue font on page 5 in the Revised Manuscript with Track Changes) .

Statistical analysis

Paragraph 1

“…is one of the most…”- review space between words

Response: The space between words here has been corrected in the Revised Manuscript with Track Changes.

Paragraph 2

“…long-acting GnRHawas analyzed…” - review space between words

Response: The space between words here has been corrected in the Revised Manuscript with Track Changes.

Results

Paragraph 1

“…Long-acting GnRHa were submitted with an increasing…”- - review space between words

Response: The space between words here has been corrected in the Revised Manuscript with Track Changes.

“A higher proportion of the reports in leuprolide (23%) and goserelin (19%) were patients of 61-80 years old, while in triptorelin (15%) and histrelin（37%）was ≤18，and patients of 19-45 years old occupied the highest proportion（37%）in buserelin reports.” - This needs to be clarified in the introduction and provide the context for the drug administration if it is condition specific.

Response: This result analysis has been explained in the blue font part on page 7 of the Revised Manuscript with Track Changes when the explanation of the difference in gender ratio of AE was carried out. Thank you.

“In terms of role code（Fig.1C）, these five drugs are essentially the primary suspect drugs in reports, except for buserelin, which was secondary suspected drugs with 100%.” - review language for clarity, claim is vague and appears to be incomplete, 100% what? Also review space

between words.

Response: To express the result of Fig.1C more clearly, we modified this sentence to“In terms of role code (Fig. 1C), these five drugs are predominantly identified as the primary suspect drugs in the reports, with the exception of buserelin, which was consistently classified as a secondary suspect drug in 100% of the cases”(marked with blue font on page 7 in the Revised Manuscript with Track Changes) . We also fixed the space problem.

“The reports of these five drugs were mostly submitted from the Americas,…” - Here are you referring to USA/ Central/South, vague word choice “Americas”.

Response: We have corrected “Americas” to “the United States” in the Revised Manuscript with Track Changes (marked with blue font on page 7).

“In terms of gender(Fig.1F), the proportion of female patients in buserelin, histrelin and triptorelin was higher than that of male patients, while the opposite results occurred on goserelin, leuprolide.” - is this context of cancer or another condition, provide specific details for results.

Response: Thank you for this insightful question. We have clarified the context and added mechanistic explanations. Please refer to Page 7 in the Revised Manuscript with Track Changes for details (marked with blue font).

“goserelin has a higher proportion (29.3%) of death,…” - review language, drug cannot have death but is the cause of death.

Response: Thank you for emphasizing the importance of precise scientific expression. We have thoroughly revised the statement to address causality attribution and strengthen methodological rigor. Please refer to Page 8 in the Revised Manuscript with Track Changes for details (marked with blue font).

“while buserelin has the largest percentage in hospitalization initial or prolonged (44.2%).” - review expression, fragmented sentence.

“The clinical outcomes of other drugs are distributed.” – vague claim, provide specific details for this claim, needs context.

Response: To solve these two unclear issues of expression, We have carefully revised the text to improve its readability and accuracy. Please refer to Page 7 in the Revised Manuscript with Track Changes for details (marked with blue font).

Figure 1 legend

“…The numbers in Fig.1B, Fig.1C, Fig.1E, Fig.1F and Fig.1G represented signal number percent.” - should be "represent"

Response: We have corrected “represented” to “represent” in the Revised Manuscript with Track Changes (marked with blue font). Please refer to Page 8 in the Revised Manuscript with Track Changes for details (marked with blue font).

Disproportionality Analysis for long-acting GnRHa - Here authors should provide specific details as to what these specific disorders were to explain the relevance/severity of the AE being

documented here. Why is this worth reporting in a publication.

Response: As we mentioned in limitations: The spontaneous reports within the FAERS database may suffer from quantitative biases and incomplete documentation. Signal analysis only represents the strength of the association between a drug and a specific adverse event detected in the self-reported data, suggesting a possible causal relationship or risk signal.

However, disproportionality Analysis plays a core role in pharmacovigilance research in the FAERS (FDA Adverse Event Reporting System) database, and its importance is mainly reflected in the following aspects: (1)Efficiently identify potential safety signals. (2)Support regulatory decisions and drug label updates. (3) Guide clinical drug use and risk stratification. (4)Promote mechanism research and biomarker development. (5) Make up for the limitations of clinical trials. (6) Promote international cooperation and data standardization.

In summary, through efficient and multidimensional signal mining, disproportionality analysis has become a bridge connecting post-marketing monitoring and precision medicine, and continues to promote the dynamic optimization of drug safety, which is worth reporting in a publication.

Paragraph 1

"With exclusion of the signals unrelated to drugs or disease progression, such as general disorders and administration site conditions, product issues, social circumstances, investigations, injury, poisoning and procedural complications, congenital, familial and genetic disorders, and surgical and medical procedures." - confused and incomplete sentence, please review, consider varying sentence structure here.

Response: In order to correct the sentence into a complete sentence, we amended it to "The signals unrelated to drugs or disease progression were excluded, such as...... (marked with blue font on page 8 in the revised manusript)"

"Excluding the specific signals (repetitive, non-SOC included, IC025 less than 0), leuprolide, goserelin, triptorelin, histrelin and buserelin had 402, 285, 152, 35 and 19 significant signals separately." - include data next to descriptor for clarity and accuracy of reporting.

Response: Thank you for your valuable feedback. To enhance clarity and accuracy, we have revised the sentence to directly associate each agent with its corresponding numerical value. The updated text now explicitly lists the number of significant signals in parentheses next to each descriptor (e.g., "leuprolide (402)"). This adjustment ensures immediate visibility of the data and aligns with your recommendation for precise reporting. The sentence that has been corrected can be found on page 8 of the Revised Manuscript with Track Changes (identified in blue).

"As shown in Fig.2 and Table S3, the most frequently reported AE for leuprolide was hot flush (n=5320), and malignant neoplasm progression(n=552) for goserelin." - this appropriate reporting style. The authors should consider reviewing and revising their results for this style.

Response: Thank you for your valuable feedback. We have carefully revised the section to improve the clarity and completeness of adverse event reporting consistent with the description of the other drugs below. Please refer to Page 8 in the Revised Manuscript with Track Changes for details (marked with blue font).

"…reported AE for both for triptorelin and buserelin, while rash was for histrelin."- review expression and grammar, verbs need to be used to report observations.

Response: We amended it to “Ovarian hyperstimulation syndrome (OHSS) was the most frequently reported AE for both triptorelin and buserelin, whereas cutaneous rash emerged as the predominant AE associated with histrelin”. Please refer to Page 8 in the Revised Manuscript with Track Changes for details (marked with blue font).

“demonstrated both a prevalence and a robust signal strength for triptorelin and buserelin.” - not sure what the authors mean here. Are you saying that this syndrome was common for both the drugs as an AE?

Response: In this case, it means high reported incidence of AE of these two drugs. To the actual incidence vs the reported incidence of AE, we made the following explanation: Firstly, our retrospective study design precludes the establishment of direct causality, which cannot be directly inferred from the observed results. The source of observed results was AE reports, which had various biases such as subjective judgments of the reporting personnel to drugs missing data, and uneven reports quality. Secondly, because the denominator was not the total number of individuals treated with an interested drug, and the numerator did not represent the overall number ofindividuals who actually experience adverse drug reactions, the ROR or IC025 values were only renderecas metric of disproportionality of AE reporting, rather than a quantifier of relative risk. However, to a certain extent, these results can provide reference for general practitioners, nursing staffand other health professionals when making clinical medical decisions.

Paragraph 2

“…triptorelin frequency with after dividing by 50),…” - review expression, confused meaning

Response: Thank you for your constructive feedback. We acknowledge the ambiguity in the original phrasing and have revised the sentence to clarify the relationship between the x-axis and the data transformation. The updated text now explicitly states: (1) which agents are included (leuprolide, goserelin, triptorelin), (2) what operation was applied to the data (division by 50), and (3) the purpose of this adjustment (visualization clarity). The revised phrasing eliminates grammatical inconsistencies (e.g., "with after dividing") and ensures logical flow. Please refer to Page 9 in the Revised Manuscript with Track Changes for details (marked with blue font).

Paragraph 3

“…long-acting GnRHa, the entire analysis results ….” – “result”, review sentence for language and grammar

Response: Thank you for your careful review. We have revised the sentence to address the grammatical and structural concerns. The sentence that has been corrected can be found on page 9 of the Revised Manuscript with Track Changes (identified in blue).

“Substantially, reproductive system and breast disorders possessed the greatest proportion in AEs of the five drugs, followed by neoplasms benign, malignant and unspecified (incl cysts and polyps),…” – delete “substantially”, incorrect use, review sentence for grammar; “incl” use complete words for academic writing.

Response: We amended it to “Reproductive system and breast disorders possessed the greatest proportion in AEs of the five drugs, followed by neoplasms benign, malignant and unspecified (including cysts and polyps), renal and urinary disorders, musculoskeletal and connective tissue

disorders and nervous system disorders (marked with blue font on page 9 in the revised manusript). ”

SAE/Important medical events linked to long-acting GnRHa

“SAE/Important medical events (IMEs) were significant factors that…” - These IME needs to be defined in the introduction to explain the relevance of the results and give clear context for the analysis.

Response: Thank you for your careful review. We explain the definition of IME later in this sentence. Please refer to Page 10 in the Revised Manuscript with Track Changes for details (marked with blue font).

“drugs.Univariate” – review spaces between words

Response: The space between words here has been corrected in the Revised Manuscript with Track Changes.

“Additionally, females exhibited a similar risk of IMEs in males.” - confused meaning, review for grammar and language.

Response: Thank you for highlighting this ambiguity. We have revised the sentence to address the grammatical and logical issues. The sentence that has been corrected can be found on page 10 of the Revised Manuscript with Track Changes (identified in blue).

“…off-label use…” - what are these, define these terms in the introduction, to given context and links for the relevance of these results.

Response: As shown in Tab.1, compared with patients receiving leuprolide, the risk of IMEs was 27.94 times higher in those administered buserelin, 6.78 times higher with goserelin, 1.22 times higher with histrelin, and 3.58 times higher with triptorelin. No significant gender-based risk disparity was observed, with females and males exhibiting comparable IME risks. Notably, patients receiving long-acting GnRHa for off-label indications (i.e., uses beyond regulatory-approved indications such as endometriosis or prostate cancer management) demonstrated 1.40-fold elevated IME risk compared to on-label applications.

“…over 80 had a approximate risk.” - not sure what the authors mean here. review for grammar and expression, clarify with specific details as to what the authors mean “approximate”.

Response: Thank you for your insightful feedback. We have revised the sentence to address the ambiguity and clarify the term "approximate". The sentence that has been corrected can be found on page 10 of the Revised Manuscript with Track Changes (identified in blue).

Discussion

Reproductive system and breast disorders

Paragraph 1 : are these a general observation, are there different observations for each of the four drugs being investigated here. Needs to clarify or provide the scope of the findings here.

Response: Thank you for highlighting the need for greater precision in describing the scope of findings. We have substantially revised this section to clarify drug-specific patterns. Please refer to Page 11 in the Revised Manuscript with Track Changes for details (marked with blue font).

“…and buserelin(n=11, ROR=2700.39), vaginal haemorrhage(n=305, ROR=3.48) for leuprolide, breast enlargemen(n=9, ROR=103.55)) for histrelin, in reproductive and breast systems.” - Confused expression, review as done for previous two drugs. Review spaces between words, review parenthesis here.

Response: We have fixed the problems with spaces and parenthesis. Please refer to Page 12 in the Revised Manuscript with Track Changes for details (marked with blue font).Thank you.

“conducted by Senra J C et al.” – include date to publication

Response: Thank you for your valuable feedback. We have now included the publication date in the reference to the study by Senra J C et al. as follows:"Senra J C et al. (2018)”. Please refer to Page 12 in the Revised Manuscript with Track Changes for details (marked with blue font).

“…completion, as compared…” – replace as with appropriate transition (when)

Response: We have changed "as" to "when" in the Revised Manuscript with Track Changes, see page 12 (marked with blue font).

“What’s more, in premenopausal women, GnRHa suppress estrogen …” – contractions are not appropriate for academic writing.

Response: Thank you for your insightful comment. We have revised the sentence to eliminate contractions and ensure formal academic language. Please refer to Page 12 in the Revised Manuscript with Track Changes for details (marked with blue font).

Paragraph 2

“Therefore, when using such drugs, it is necessary to strictly monitor whether the indicators of reproductive and breast system are normal.” - Is there data to report whether there are dose specific differences in these observations?

Response: In our study, there were many missing items on dose information in adverse events reporting for other four drugs except leuprolide, so dose-specific differences in observed adverse events have not been examined. In the future, further studies of dose-related adverse event rates are warranted.

Neoplasms benign, malignant and unspecified (incl cysts and polyps): Are these reversible changes? Here does dosage and frequency impact the outcomes. Does the author have data/statistical evidence to report this claim.

Response: As we mentioned in limitations: The spontaneous reports within the FAERS database may suffer from quantitative biases and incomplete documentation. A large part of the data on the route and dosage of the five drugs is missing or unknown. Therefore, we believe that these data cannot support whether the route of administration or dose is associated with the reported AE. However, our future plan is to investigate this issue through meta-analysis or clinical data. Thank you for pointing out the direction for our future research. We have also added future research plans to conclusions. Please refer to Page 18 in the Revised Manuscript with Track Changes for details (marked with blue font).

Paragraph 1

"…unspecified (incl cysts and polyps) induced by leuprolide, goserelin and triptorelin, we consider that it is highly correlated…" – "incl" is unclear abbreviation, "we" pronouns are not appropriate for academic reporting, "is", delete; review for grammar and language

"…in the three drugs with 59.60%,39.37% and 22.88% severally." – incorrect use of "severally"

Response: Thank you for your meticulous feedback. We have revised the sentence to address your concerns. Please refer to Page 12 in the Revised Manuscript with Track Changes for details (marked with blue font).

Musculoskeletal and connective tissue disorders

"In musculoskeletal and connective system of quite a few reports." – vague, review language and expression.

Response: Thank you for your critical feedback. We have thoroughly revised the sentence to eliminate vagueness and improve academic precision. Please refer to Page 13 in the Revised Manuscript with Track Changes for details (marked with blue font).

"Our research showed that bone pain was the most common AE for both goserelin and leuprolide, epiphyses premature fusion for triptorelin, muscular weakness for buserelin, and growth accelerated for histrelin in musculoskeletal and connective tissue disorders." - premature fusion of epiphysis (review spelling and expression).

Response: We have revised "epiphyses premature fusion" to "premature fusion of epiphysis". Please refer to Page 13 in the Revised Manuscript with Track Changes for details (marked with blue font). Thanks a lot.

Nervous system disorders

"…respectively was neuropathy peripheral (n=50, ROR=2.55), dementia alzheimer's type (n=106, ROR=81.06), embolic stroke (n=29, ROR=3.95). Nevertheless, no adverse events associated with this system were reported in buserelin and histrelin in our statistics." – "neuropathy peripheral", peripheral neuropathy, adjective needs to come before the noun; "dementia alzheimer's type" see previous comment, adjective needs to come before the noun; "our statistics" Should be results.

Response: We have revised "neuropathy peripheral" to "peripheral neuropathy", "dementia alzheimer's type" to "alzheimer's type dementia". Please refer to Page 13 in the Revised Manuscript with Track Changes for details (marked with blue font). Thanks a lot.

Psychiatric disorders

Paragraph 1

"In the instructions for this type of drugs, such adverse reactions are more common but less serious." – vague expression, not cleat what the author referring to here, review to be more specific here.

Response: Thank you for your critical feedback. We have thoroughly revised the sentence to eliminate vagueness and improve academic precision. Please refer to Page 13 in the Revised Manuscript with Track Changes for details (marked with blue font).

"In our study, except for buserelin, the other four drugs involved only mood swings, mood altered

events.” – review for language and clarity.

Response: Thank you for your constructive feedback. We have revised the sentence to enhance clarity and precision. Please refer to Page 13 in the Revised Manuscript with Track Changes for details (marked with blue font).

Paragraph 2

“Frokjaer VG et al. suggested” – include date for publication

Response: We have now included the publication date in the reference to the study by Frokjaer VG et al. as follows: "Frokjaer VG et al. (2020)”. Please refer to Page13 in the Revised Manuscript with Track Changes for details (marked with blue font).

“…that the transition of sex hormones…” - not clear what the author is referring to here. In what way are the hormones transitioning.

Response: We have included an explanation in this sentence, which is due to sharp decreases in estradiol levels. Please refer to Page 13 in the Revised Manuscript with Track Changes for details (marked with blue font).

“… during short-term manipulation…” – would this be increase/decrease in the levels of hormones/ dose dependent effects?

Response: As we mentioned in limitations: The spontaneous reports within the FAERS database may suffer from quantitative biases and incomplete documentation. A large part of the data on the route and dosage of the five drugs is missing or unknown. Therefore, we believe that these data cannot support whether the route of administration or dose is associated with the reported AE. However, our future plan is to investigate this issue through meta-analysis or clinical data. Thank you for pointing out the direction for our future research. We have also added future research plans to conclusions. Please refer to Page 18 in the Revised Manuscript with Track Changes for details (marked with blue font).

Paragraph 3

“However, we were surprised to find …” - avoid emotive language in reporting to prevent bias and to be objective in reporting.

“… GnRHa with hormone addback…” - not clear what this phrase means here

“SSRIs” - provide description for abbreviation

“ Wagner-Schuman M et al. demonstrating its favorable mood effects[28].” – include date for publication, What does the authors mean by favourable, provide specific details to support the claims.

Response: Thank you for emphasizing the importance of objective language. We have integrated the above several problems and made modifications. The subjective emotion in the expression is eliminated, the date of reference study is added, and SSRIs and PMDD are explained respectively. In addition, we want to explain that "GnRHa with hormone addback" is derived from the reference and is considered as a treatment for PMDD. After adjusting the structure and expression of the sentence, the favorable mood effect of GnRHa was also revealed: GnRHa with hormone addback

should be considered as a treatment for premenstrual dysphoric disorder (PMDD). Please refer to Page 14 in the Revised Manuscript with Track Changes for details (marked with blue font).

Paragraph 4
“psychiatric” – term is in bold, unclear why
Response: This is our mistake, we have removed the bold.

Skin and subcutaneous tissue disorders
Paragraph 1
“…skin-related adverse reactions, such as estrogen deprivation symptoms and oily skin…” – is this a reference to endogenous and or exogenous estrogen levels, provide specific details as to how this supports the findings of this paper.
Response: The original literature does not deeply discuss whether this is endogenous or exogenous. Therefore, in order to avoid misleading readers, this section does not explain in detail the question of endogenous or exogenous hormone levels. Thank you.

“…erythema nodosum[32] , drug-induced vasculitis…” – include “and at the end of list
Response: We added "and" to the two nouns as a connection. Please refer to Page 14 in the Revised Manuscript with Track Changes for details (marked with blue font).

Paragraph 2
“The previous of our results show that histrelin…” - review expression for grammar and language
Response: Thank you for your meticulous feedback. We have revised the sentence to address grammatical inaccuracies and improve clarity. Please refer to Page 14 in the Revised Manuscript with Track Changes for details (marked with blue font).

“By the same coincidence, no adverse events associated with this system …” - review for language and expression
Response: Thank you for your insightful critique. We have revised the sentence to enhance clarity and academic rigor. Please refer to Page 15 in the Revised Manuscript with Track Changes for details (marked with blue font).

“If patients suffer from subcutaneous nodules, urologists should consider changing the drug to another types of luteinizing hormone-releasing hormone analogues such as goserelin acetate.” - should be type (singular), is there literature to support this finding/general practice/recommendation?
Response: We have revised “types” to “type” (marked with blue font). Thank you.

Gastrointestinal disorders
“Ohlsson B et al. reported a case of a 30-year-old woman who developed CIPO as a result of buserelin-induced formation of anti-GnRH antibodies leading to destruction of GnRH-producing neurons in the myenteric plexus.[36]. - There needs to be this type of reporting to given context

for these observations as much of this cannot be blanket AEs.

Response: Thank you for raising this important point. We have revised the text in the gastrointestinal disorders section of the discussion on page 15 to clarify that the Ohlsson B et al. (2007) case describes a specific immunogenic mechanism linked to buserelin (a GnRH agonist), rather than a class-wide adverse effect. Key modifications include: (1) Explicitly framing the case as a rare, antibody-mediated event distinct from general GnRH analog-associated gastrointestinal effects. (2) Adding the caveat that such antibody formation "has not been widely documented across the GnRH analog class." (3) Structuring the narrative to contrast isolated severe complications (CIPO/ED) with emerging therapeutic benefits in IBS, emphasizing that these represent distinct biological pathways. This preserves the case report's scientific relevance while preventing overgeneralization of its findings.

"However, the case of their beneficial effects on the gastrointestinal tract deserves our attention. Gondotropin-releasing hormone agonist administration is effective in…" - avoid such superficial and emotive claims as it detracts from the reported results.

Response: Thank you for your critical feedback. We have revised the text to eliminate subjective language and strengthen scientific rigor. Please refer to Page 15 in the Revised Manuscript with Track Changes for details (marked with blue font).

- is there a particular drug type that is recommended and what is the recommended or therapeutic dosage in the treatment of this group of disorders. This information would add value to the manuscript and also some context for these reporting.

Response: As we mentioned in limitations: The spontaneous reports within the FAERS database may suffer from quantitative biases and incomplete documentation. A large part of the data on the route and dosage of the five drugs is missing or unknown. Therefore, we believe that these data cannot support whether the route of administration or dose is associated with the reported AE. However, our future plan is to investigate this issue through meta-analysis or clinical data. Thank you for pointing out the direction for our future research. We have also added future research plans to conclusions. Please refer to Page 18 in the Revised Manuscript with Track Changes for details (marked with blue font).

Vascular disorders: vague reporting, provide some details to specify the severity of these AEs

Paragraph 1

"flush, in fact, also applies to goserelin, histrelin and triptorelin of vascular disorders." - review for grammar and expression.

Response: Thank you for your meticulous critique. We have revised the sentence to address grammatical inaccuracies and improve clarity. Please refer to Page 15 in the Revised Manuscript with Track Changes for details (marked with blue font).

"venous thrombosis limb," " shock haemorragic"– review expressions

Response: "Venous thrombosis limb," "shock haemorragic" heve been amended to "venous limb thrombosis" and"haemorrhagic shock "respectively. Please refer to Page 16 in the Revised Manuscript with Track Changes for details (marked with blue font).

Paragraph 2

“disturbances that confer increased risk of (CVD) diabetes mellitus (DM) and cardiovascular disease” –“ confer”, review word choice; “increased risk of (CVD)” , provide description for abbreviation and incorrect use of parenthesis; “and cardiovascular disease”, confused use here.

Response: We have revised “confer” to“generate”. In addition, the full names and abbreviations of DM and CVD have also been corrected. Please refer to Page 16 in the Revised Manuscript with Track Changes for details (marked with blue font).

Paragraph 3

“As for the principle” – confused, not sure what the authors mean here.

Response: We have revised“As for the principle”to “Regarding why the use of GnRHa is more likely to cause vascular disorders”. Please refer to Page 16 in the Revised Manuscript with Track Changes for details (marked with blue font).

“Dockery F et al. once gave an explanation that loss of androgens…” – include date for publication, is this finding relevant to current practice?

Response: Date has been added in the Revised Manuscript with Track Changes. This finding relevant to current practice. As shown in the revised content of the previous question, this earlier study could explain how the use of GnRHa causes vascular disorders.

“…adversely affect cardiovascular risk twenty years ago[2, 43].” – revise for language and expression

Response: In order to more clearly express our discussion of the evidence, we brought this adverb of time (more than twenty years ago) ahead of the researchers. Please refer to Page 16 in the Revised Manuscript with Track Changes for details (marked with blue font).

Paragraph 4

“Therefore, the cardiovascular harm of GnRHa is also not to be underestimated” - revise for language and expression

Response: Thank you for your critical feedback. We have revised the text to eliminate subjective language and strengthen scientific rigor. Please refer to Page 16 in the Revised Manuscript with Track Changes for details (marked with blue font).

“prescribing HT especially” - provide a detailed description of the abbreviation at first use.

Response: We have revised “HT、ADT、CAD” to address grammatical inaccuracies and improve clarity. “HT” was replaced by “hypertension”, and “ADT , CAD” were explained in English with their full names. Please refer to Page 16 in the Revised Manuscript with Track Changes for details (marked with blue font).

SAE/Important medical events

“buserelin, they took” – review space between words

Response: The space between words here has been corrected in the Revised Manuscript with Track Changes.

Limitations

“2) The ROR solely signifies an elevated risk of reported AEs and does not mirror the actual clinical risk.” - This could have been discussed more explicitly in the results and discussion.

Response: This is one of the acknowledged limitations of the FAERS database. Therefore, we have deliberately arranged this paragraph to describe it.

“(4) The absence of exposure data precludes the calculation of morbidity and mortality rates.” - review for language and expression

Response: Thank you for your insightful feedback. We have revised the text to enhance clarity and methodological rigor. Please refer to Page 17 in the Revised Manuscript with Track Changes for details (marked with blue font).

(6) There may be differences in the timing of the launch of the five drugs, so there may also be differences in the reporting of adverse events as a result.”- a brief on the timeline for the introduction of the drugs into clinical practice is relevant to this paper could be included in the introduction.

Response: Thank you very much for this meaningful suggestion. We have described the time they were first approved in the introduction on page 3 of the Revised Manuscript with Track Changes (marked with blue font).

Conclusion

“In our study, 43683, 5138, 3143, 501, and 52 reports of leuprolide, goserelin, triptorelin, histrelin and buserelin were submitted to FAERS up to September 2023. Ultimately, a comprehensive analysis and comparative assessment of 402, 285, 152, 35 and 19 significant signals for the five long-acting GnRHa were conducted.” - put data next to drug for clear reporting

Response: Thank you for your insightful feedback. We have revised the text to enhance clarity and methodological rigor. Please refer to Page 17 in the Revised Manuscript with Track Changes for details (marked with blue font).

“A better illustration of what an actual pharmacovigilance research entails was provided by this.”- vague claim

Response: We have revised the text to enhance clarity with this sentence changed to “The results of our analysis better illustrate the importance of actual pharmacovigilance studies”. Please refer to Page 17 in the Revised Manuscript with Track Changes for details (marked with blue font).

“actions. As a result, drug safety management should consider the disease , the drug itself (route of use), age and other factors, so as to monitor the drug according to these characteristics.” – vague claim as route of drug administration was not reported here and should have been part of the results and discussion to provide relevant context and demonstrate robust analysis here.

Response: The phrase "route of administration" has been added to the original sentence. Please refer to Page 18 in the Revised Manuscript with Track Changes for details (marked with blue font).

Journal Requirements:

Please review your reference list to ensure that it is complete and correct. If you have cited papers that have been retracted, please include the rationale for doing so in the manuscript text, or remove these references and replace them with relevant current references. Any changes to the reference list should be mentioned in the rebuttal letter that accompanies your revised manuscript. If you need to cite a retracted article, indicate the article's retracted status in the References list and also include a citation and full reference for the retraction notice.

Response: No Retractions found matching all the references in our manuscript.

Additional Editor Comments:

The following are suggestions to address minor issues in the manuscript submitted by Huancun Feng.

• If applicable, we recommend that you deposit your laboratory protocols in protocols.io to enhance the reproducibility of your results. Protocols.io assigns your protocol its own identifier (DOI) so that it can be cited independently in the future. For instructions see: https://journals.plos.org/plosone/s/submission-guidelines#loc-laboratory-protocols. Additionally, PLOS ONE offers an option for publishing peer-reviewed Lab Protocol articles, which describe protocols hosted on protocols.io. Read more information on sharing protocols at https://plos.org/protocols?utm_medium=editorial-email&utm_source=authorletters&utm_campaign=protocols.

Response: Thank you for your suggestion regarding protocol deposition. As our study is a computational analysis/database mining project without laboratory-based experiments, all methodological details (including data sources, analytical pipelines, and code availability) have been thoroughly described in the manuscript's materials and methods.

To enhance reproducibility, we have provided full access to the datasets used (OpenVigil 2.1 website: http://openvigil.sourceforge.net/) and the query path was introduced in detail. Since no laboratory protocols were involved, deposition to protocols.io is not applicable in this case. However, we confirm that the study design and analytical workflows adhere to FAIR principles (Findable, Accessible, Interoperable, Reusable).

Please let us know if further clarifications are needed.

• In the previous version, the authors did not include the figures, table, or supplementary material. Please submit a revised version that includes all necessary components to allow proper evaluation of the manuscript.

Response: We have re-examined and ensured the upload of figures and other necessary documents.

• In the following sentences, it should be clearly stated that the adverse events (AE) were observed in association with specific GnRH analogues. For example: "The AEs with the strongest signal intensities for leuprolide, goserelin, triptorelin, histrelin, and buserelin were urinary tract toxicity (ROR = 490.89), malignant spinal cord compression (ROR = 3958.65), ovarian hyperstimulation syndrome (ROR = 719.85), delayed menarche (ROR = 16901.8), and noninfective oophoritis (ROR = 26614.6), respectively." Based on the data presented in Table S3, each of these ROR values corresponds to only one GnRHa. Please revise this paragraph to explicitly associate each AE and ROR with the specific GnRHa in which it was observed.

Response: Thank you for your careful review. We have revised this paragraph to explicitly associate each AE and ROR with the specific GnRHa, as shown with the purple font section on page 9 in Revised Manuscript with Track Changes.

• In Figure 2, the authors state that the top 20 AEs are shown for each agonist. However, some graphs display fewer than 20 AE. Please clarify this discrepancy in the text or adjust the figures to consistently display 20 AE for each GnRHa.

Response: The bubble plot presents adverse events (AEs) derived by combining the top 20 AEs ranked by case count and the top 20 AEs ranked by ROR value, followed by deduplication based on AE name. Some AEs did not meet the "signal" threshold and thus have fewer than 20 signals.

• Define "SAE" in the title of the Results section: "SAE/Important Medical Events Linked to Long-Acting GnRHa."

Response: We have revised "SAE" to "Serious adverse events", as shown with purple font section in subheadings of the results and the discussion in Revised Manuscript with Track Changes.

• The following sentence needs style harmonization: "Besides local reactions, systemic hypersensitivity reactions such as urticaria, anaphylaxis, serum sickness, and Henoch-Schönlein purpura (HSP) have been reported during gonadotropin-releasing hormone (GnRH) analogue treatment in Central Precocious Puberty[39]. Most of these adverse events are associated with intramuscular injections." To maintain consistency throughout the document, remove the full hormone name and retain only the abbreviation "GnRH."

Response: We have removed the full hormone name and retain only the abbreviation "GnRHa", as shown with the purple font section on page 15 in Revised Manuscript with Track Changes.

• If buserelin shows the lowest number, frequency, and intensity of AEs, please explain why this GnRHa is associated with the highest number of Important Medical Events (IME). Additionally, clearly define which criteria were used to classify events as IME or what was consider as IME in your analysis.

Response: 1. We sincerely appreciate your insightful observation regarding the disparity between buserelin's overall adverse event profile and its association with Important Medical Events.

2. Our explanations for the observed discrepancy:

(1) Severity vs. Frequency: While buserelin demonstrated fewer total AEs, its IMEs (e.g., ascites, pulmonary fibrosis) were rare but clinically significant. This aligns with pharmacovigilance data showing that certain GnRH agonists may trigger acute immune-mediated reactions despite overall tolerability.

(2) Reporting Bias: IMEs (e.g., hospitalizations) are more rigorously documented in databases compared to mild AEs, potentially amplifying their relative representation.

(3) We have added the following contents in the section of Serious adverse events/Important medical events in the discussion (marked with purple font) "The higher IME rate with buserelin, despite its favorable overall AE profile, underscores the importance of severity-based drug safety assessments. Clinicians should consider both AE frequency and potential for rare but catastrophic events, particularly in high-risk populations (e.g., patients with autoimmune histories)".

3. Definition of IMEs in our study has been noted on page 11 (marked with blue font) " An

Important Medical Event (IME) is a clinical event that does not meet the stringent criteria for a serious adverse event (SAE) but has the potential to be clinically significant and requires proactive medical intervention to avoid a deterioration or serious outcome (such as hospitalization, disability, or death). IME is also a document in the form of a list of updated versions regularly published on EMA's official website (https://www.ema.europa.eu/en/human-regulatory/research-development/pharmacovigilance/eudravigilance/eudravigilance-system-overview)" . The definition of IME is officially unified, rather than determined by our subjective analysis.

• In the conclusion, the authors do not indicate, based on their findings, which GnRHa might be considered the most suitable pharmacological option. In my opinion, it would be appropriate to at least suggest a preferred candidate based on the observed data.

Response: We appreciate the reviewer's constructive suggestion regarding the need to clarify the clinical implications of our findings.

However, our data cannot universally recommend a single "best" option across all contexts. We can just propose scenario-based recommendations based on observed efficacy-safety trade-offs: For indications requiring long-term suppression (e.g., hormone-sensitive cancers); Leuprolide or histrelin may be preferred due to their sustained efficacy and lower IME risk; For patients with comorbidities (e.g., cardiovascular disease), triptorelin's neutral metabolic profile might be optimal.

Therefor, the choice of which drug to use is more inclined to be based on indications clinically, while our research is merely from the perspective of reminding of safety. FAERS, as an observation database for discovering potential medication risks, should be used as a monitoring tool for adverse reactions of clinical medication. Its observational study cannot yet override indication-specific RCT evidence.

• While revising your submission, please upload your figure files to the Preflight Analysis and Conversion Engine (PACE) digital diagnostic tool, https://pacev2.apexcovantage.com/. PACE helps ensure that figures meet PLOS requirements. To use PACE, you must first register as a user. Registration is free. Then, login and navigate to the UPLOAD tab, where you will find detailed instructions on how to use the tool. If you encounter any issues or have any questions when using PACE, please email PLOS at figures@plos.org. Please note that Supporting Information files do not need this step.

Response: Thank you for your guidance regarding figure file processing. We have carefully reviewed the PLOS requirements and completed the steps. We have completed the PACE registration and upload. All figure files have been successfully uploaded to the PACE platform (https://pacev2.apexcovantage.com/) for preflight analysis.The tool confirmed compliance with PLOS formatting standards (e.g., resolution, file type, and color mode). And revised figures are attached to the submission in the designated "Figures" section.

We confirm that all figures meet PLOS guidelines based on PACE diagnostic results. Should any further adjustments be needed, we will promptly address them. Please let us know if additional information is required. We appreciate your assistance throughout the submission process.

We believe that these revisions have significantly improved the quality and depth of our discussion section. However, we welcome any further suggestions or comments you may have on this or any other aspect of our manuscript.

Finally, I would like to express my sincere gratitude once again to the reviewers and editors of your journal for their diligent efforts and insightful feedback. Your comments are invaluable to me, as they not only aid in improving the quality of the manuscript but also points out the direction for my future research. I hope that these revisions will further align with the publication requirements of the journal and I look forward to continued communication with both the reviewers and the editorial team. If you have any further suggestions or require acess to the full text of the Revised Manuscript with Track Changes, please do not hesitate to reach out. I would be more than happy to provide it.
Thank you once again for your thoughtful review and valuable opinions!
